# Role of gamma-irradiated sodium alginate on growth, physiological and active components of iceberg lettuce (*Lactuca sativa*) plant

**DOI:** 10.1186/s12870-024-04853-8

**Published:** 2024-03-13

**Authors:** Amina A. Aly, Noha E. Eliwa, Gehan Safwat

**Affiliations:** 1 Natural Products Research Department, National Center for Radiation Research and Technology, Egyptian Atomic Energy Authority (EAEA), Cairo, Egypt; 2 Faculty of Biotechnology, October University for Modern Sciences and Arts (MSA), Giza, Egypt

**Keywords:** Sodium alginate. Gamma ray. Chemical content. Lettuce

## Abstract

**Background:**

One of the most widely recognized biostimulators of plant development; is oligoalginate, which regulates the biological processes of plants and was used in horticultural fields as a plant growth regulator. The plan of the current research was to study, however, the foliar application of un-irradiated and irradiated Na-alginate (UISA and ISA) to improve the growth, physiological activity, and other active components of the Egyptian iceberg lettuce plant. Degraded Na-alginate is equipped with exposure of sodium alginate in its solid state to gamma-rays at different dose levels (0.0, 25, 50, 75, and 100 kGy). The characterization of the oligo-alginates achieved by γ-radiation deprivation at different dose levels was performed by FTIR, XRD, TGA, SEM, and TEM. Different concentrations of irradiated sodium alginate at dose levels of 100 kGy (200, 400, 600, and 800 ppm, as well as deionized water used as a control) were sprayed with a hand sprayer every week after transplanting the iceberg lettuce seedlings in the field until the harvest stage. Morphological traits were evaluated, as well as pigments, ascorbic acid, phenols, flavonoids, soluble proteins, and antioxidant activity.

**Results:**

Irradiated Na-alginate resulted in the depolymerization of Na-alginate into small molecular-weight oligosaccharides, and the best dose to use was 100 kGy. Certain chemical modifications in the general structure were observed by FTIR analysis. Two absorbed bands at 3329 cm^−1^ and 1599 cm^−1^, were recognized that are assigned to O–H and C-O stretching, respectively, and peaks achieved at 1411 cm^−1^ represent the COO-stretching group connected to the sodium ion. The peak obtained at 1028 cm^−1^ was owing to the stretching vibration of C-O. The results of TGA provided that the minimum weight reminder was in the ISA at 100 kGy (28.12%) compared to the UISA (43.39%). The images of TEM pointed out that the Na-alginate was globular in shape, with the particle distribution between 12.8 and 21.7 nm in ISA at 100 kGy. Irradiated sodium alginate caused a noteworthy enhancement in the vegetative growth traits (leaf area, stem length, head weight, and leaf number). By spraying 400 ppm, ISA showed a maximum increase in total pigments (2.209 mg/g FW), ascorbic acid (3.13 mg/g fresh weight), phenols (1.399 mg/g FW), flavonoids (0.775 mg/g FW), and antioxidant activities (82.14. %). Also, there were correlation coefficients (R values) between leaf area, stem length, head weight, and leaf number values with total pigment content, antioxidant activity, total soluble proteins, and ascorbic acid.

**Conclusions:**

The outcomes of the recent investigation demonstrated that the application of spraying irradiated Na-alginate (100 kGy) resulted in an improvement of the considered characters.

## Background

Currently, the emphasis in scientific advancement on crop biostimulants to improve expected plant processes, that consequently increase nutrient absorption, efficiency, crop value, and plant production, as well as improved plant performance against biotic and abiotic stresses, is principal for sustainable agriculture production and ensuring food safety in climatic change [1, 2]. Recently, many polysaccharides support products have been arranged and incorporated for numerous purposes, including biomedical, environmental, and technical appliances [3–5]. Polysaccharides derived from algae are well-liked biostimulators that have several impacts on plants [6]. They are inexpensive, physiologically reactive, biodegradable, biocompatible, and non-poisonous [7]. Different exogenous as well as endogenous aspects regulate plant growth, development, and production in both direct and indirect ways. Radiation-derived low-molecular-weight oligomers of natural polysaccharides (like Na-alginate, carrageenan, and chitosan) have been shown to perform as plant growth-promotor agents and are acting in the same way as the PGPS [8–10]. Sodium alginate is an eco-friendly polysaccharide produced from brown algae that is widely accessible and extensively utilized as a development-promoting agent in its gamma-irradiated shape. Alginate is broken down in different ways by ultrasonic, ultraviolet, and gamma-rays. Gamma-ray is considered one of the most efficient degradation processes. Alginate depolymerization by irradiation doesn't require special management of temperature, environment, or additives [11]. The natural polysaccharides; carrageenan, Na-algenate, and chitosan are broken down by gamma into tiny oligomers with relatively small molecular weights [12]. Irradiated Na-alginate (ISA) is used as a plant development promoter as well as a biofertilizer [13]. Unlike traditional deprivation procedures that are highly complex and time-consuming, radiolysis break-down proposes a one-step effervescent and additive-free technology for large-scale manufacture of value-added oligomers. Furthermore, there is constant concern about the safety of utilizing chemically treated polysaccharides, especially for food products. One of the main advantages of radiolytic deprivation of polysaccharides is that it may produce quantifiable and reproducible changes without requiring a special mechanism to modify the temperature, environment, and additives [14, 15]. In the same regard, sodium alginate is considered one of the most commonly applied polymers in the agriculture industry, owing to its physio-chemical traits, water holding capability, softness, and non-toxic process. It also offers improved handling, less dustiness, flow ability, a long service life, and improved soil formation [2]. Moreover, its deprivation substances were found to manipulate the plant’s physiological activity, like elicitors, enhance germinating, shoot development, and root growth [16]. Alginate's great flexibility and qualities, lack of toxicity, and compatibility with several components make it an indispensable polymer in the agriculture sector and compatibility with several components. It is utilized by broad profits as a medium for impotent microorganisms. It can be applied in the form of capsules through crop growth, giving improvements in yields, value, and pest control, thus supporting sustainable agricultural methods that boost agricultural output, reduce the use of agrochemicals, and have fewer harmful effects on the environment [17]. The application of ionizing radiation to breakdown this naturally bioactive compound and then apply it as plant promoter agents is a novel technology technique that maximizes the genetic potential of plants in terms of development, yields, and value [18]. Gamma-ray radiolysis offers a clean, one-step technique for the production of less molecular-weight oligomers of Na-alginate [19, 20], compared with conservative methods like acid–base hydrolysis and enzymatic techniques [21]. Application of these oligomers in crops have been shown to promote general plant growth, seed germination, shoot elongation, root growth, flower production, photosynthetic capacity, enzyme activities, phytoalexin induction, etc. [22]. *Lactuca sativa* is an annual plant belonging to the Asteraceae family and is an important leafy vegetable mostly used fresh as a salad or in salads mixed with other types of fresh vegetables. Also, it is one of the most consumed fresh-cut vegetables, attributed to its crispy texture and fresh appearance, as well as its healthy contents of phytochemicals like phenolic compounds, ascorbic acid, and carotenoids [23]. It is considered the most significant vegetable in the leafy vegetables group, and due to its economic significance, lettuce is widely utilized in environmental bioassay. This is because of its high sensitivity, rapid germination, homogeneous germination, low cost, absence of expensive equipment, and high accessibility [24].

Within this context, it is suggested to investigate the categories of gamma-irradiated Na-alginate (ISA) that may be performed to enhance the growth, physiological activities, yield attributes, and improve plant metabolic production.

## Results and discussion

Low molecular weight sodium alginate was prepared by subjecting crude Na-alginate to gamma irradiation at different dose levels. The molecular weight of sodium alginate mainly has vital properties when considered to apply in various fields. Different dosages of γ- irradiation resulted in the breaking down of Na-alginate into small molecules [25]. The molecular weight of sodium alginate is decreased into smaller ones, and this reduction is due to the depolimerization of the sodium alginate polymer obtained by gamma irradiation. The characterization of UISA and ISA was performed with the following methods: viz. FTIR, XRD, TGA, SEM, and TEM.

### Fourier transform infra red (FTIR)

**Fig. 1:**
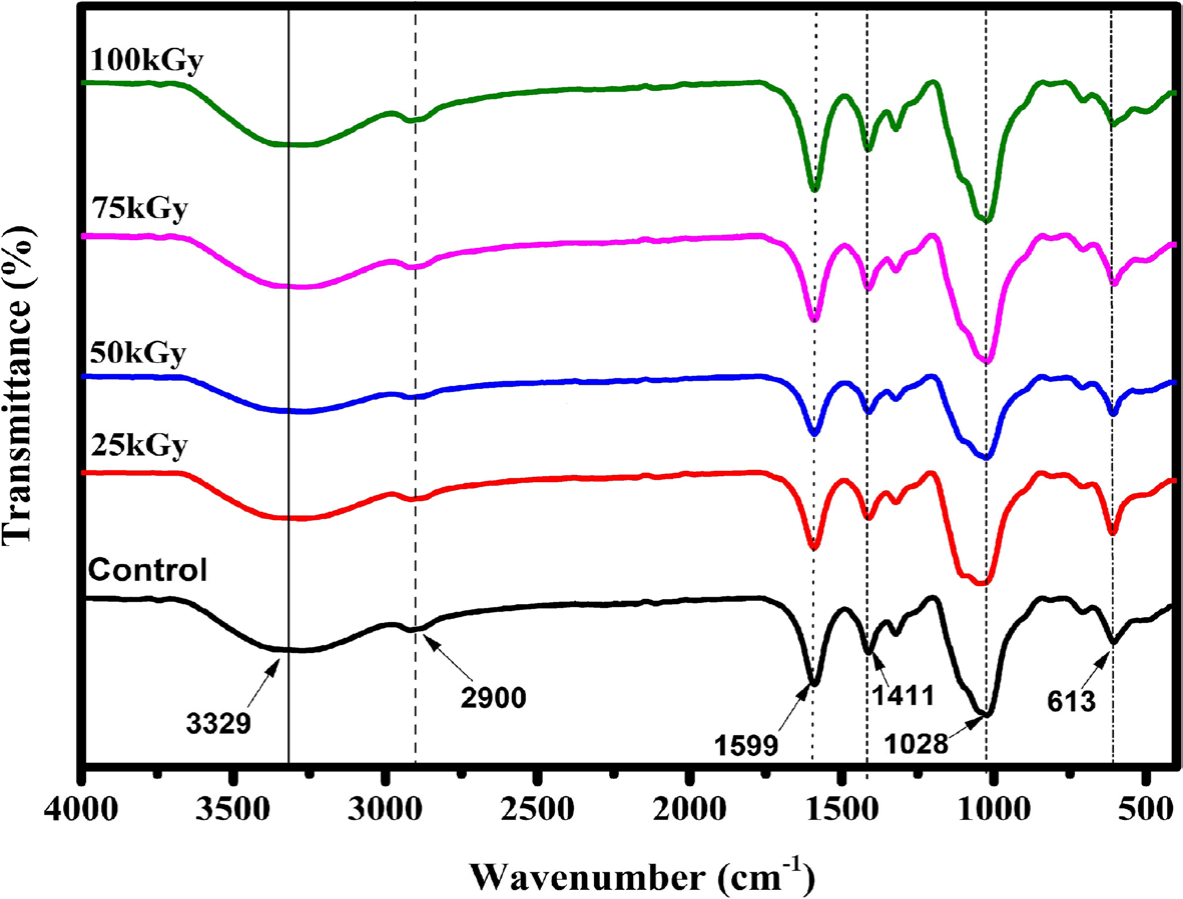
Fourier transforms infrared (FTIR) spectra of un-irradiated and γ-irradiated sodium alginate at different dose levels

The FTIR spectroscopic analysis was determined to prove that alginate undergoes chemical alteration later than the irradiation process. The peak differences were observed in the region of 4000–400 cm^−1^ of UISA and ISA at doe levels of 25, 50, 75, and 100 kGy, respectively, which are provided in Fig. 1. The backbone construction of SA was detected to stay intact after γ-irradiation; however, certain chemical alterations in the general construction were seen. Absorbed bands at 3329 cm^−1^ and 1599 cm^−1^, were detected that are assigned to O–H and C-O stretching, respectively, and peaks achieved at 1411 cm^−1^ represent the COO-stretching group connected to the sodium ion. The peak recognized at 1028 cm^−1^ is attributed to the stretching pulsation of C-O. Thus, the spectrum exposed the development of fresh carboxyl and hydroxyl groups. This suggests that γ-irradiated samples under severe conditions might be causing glycosidic bond cleavage by the changes in the structure of the reducing terminal end residues. The FTIR analysis revealed that the backbone construction of SA was detected to be unaltered after γ-irradiation; however, certain chemical changes were seen in the overall structure. The identical FTIR spectra of UISA and ISA show that there were no significant alterations were observed, indicating that the constancy of the major polysaccharide chain structurally stayed the same through irradiation-induced deprivation [26]. The development of additional carboxyl and hydroxyl groups owing to the split of glycosidic bonds [27]. Moreover, there are two peaks at 1632 and 1414 cm^−1^ that point to the carboxyl stretching groups linked to the sodium ion. This is induced by the deformation stretching pulsation of the carboxylate for the previous step, while the last is attributed to the hydroxylic asymmetric pulsation by contributing to the carboxylic formation stretching pulsation of the carboxylate group. As reported previously, irradiation produces scission of the 1–4 glycosidic bonds of polysaccharides [11]. In the same concern, the FTIR spectra obtained for un-irradiated and irradiated sodium alginate show that no new efficient group peaks are induced for γ-irradiated sodium alginate, suggesting that only the polymer is broken down into small molecules but the main unit structure is not broken [25].

### X-Ray diffraction (XRD) analysis

**Fig. 2:**
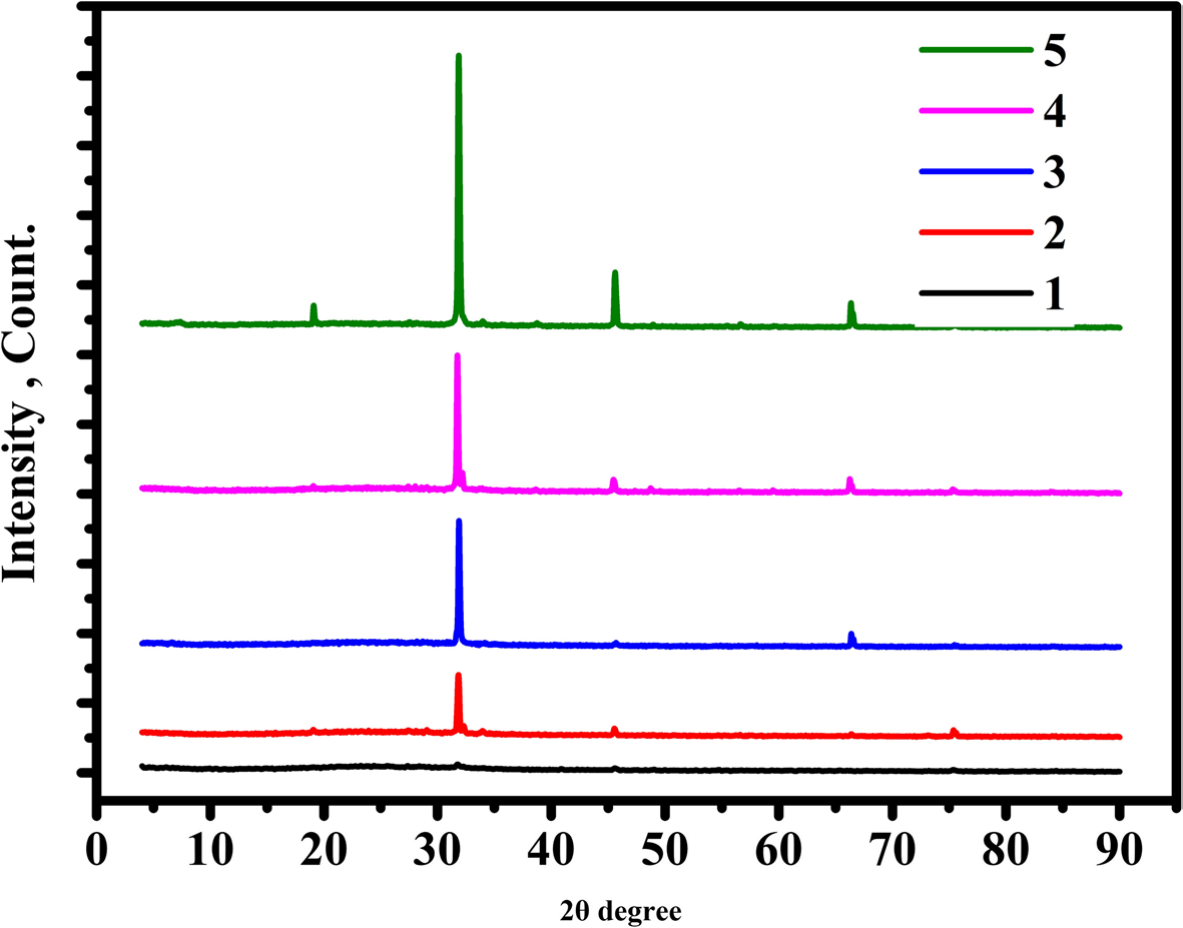
X-Ray diffraction (XRD) spectrum of un-treated and irradiated sodium alginate. 1; control, 2; 25 kGy, 3; 50 kGy, 4; 75 kGy and 5; 100 kGy

The XRD analysis is carried out to evaluate the crystallinity and structural changes of sodium alginate before and after being irradiated. Results provided in Fig. 2 and Table 1 show the XRD patterns of UISA and ISA exposed to γ-irradiation dose levels of (25, 50, 75, and 100 kGy) in addition the un-irradiated Na-alginate is used as control. The XRD patterns of the obtained ISA showed new strong peaks at 2θ = 15.6°, 31.8°, 45.5°, and 65.4° and the strength of the peaks is enhanced by rising γ-irradiation dose levels. Also, the Full Width at Half Maximum (FWHM) decreased by increasing the irradiation dose level from 0.210º for 25 kGy to 0.163 for the irradiation dose level of 100 kGy. This indicates that γ-irradiation procedure is the reason for their break-down and destruction of sodium alginate structurally. The degradation was most likely caused by the direct effects of radiation and the indirect effects of the oxidation procedure. When polysaccharide is exposed to irradiation, it results in the breakdown of the orderly structure of the intermolecular hydrogen bond. Therefore, the stiffness of the chain is affected by the intramolecular hydrogen bond, which reduces the level of the materials crystallinity. Moreover, diffraction peaks at 2Ɵ = 12.78^°^, 13.28°, 14.32°, 18.10°, 21.41°, 22.54°, 24.77°, 28.71°, and 38.02° correspond to the semi-crystalline structure of sodium alginate [28]. It was supposed that the degradation primarily happened effectively in the amorphous regions, and after that, by increasing irradiation doses, it performed extremely reasonably from the edges to the insides of the crystalline [29].

**Table 1:** Crystalline parameters of the un-irradiated and γ-irradiated Na-alginate

Sample	d, nm	FWHM, degree	2θ, degree	L_θ_, nm
Control	-	-	-	-
25 kGy	0.281128	0.20970	31.8053	63.74921
50 kGy	0.280606	0.13090	31.8660	63.3763
75 kGy	0.281838	0.12950	31.7230	63.08982
100 kGy	0.280875	0.1629	31.8347	50.69253

Full Width at Half Maximum (FWHM)

### Thermogravimetric analysis (TGA)

**Fig. 3:**
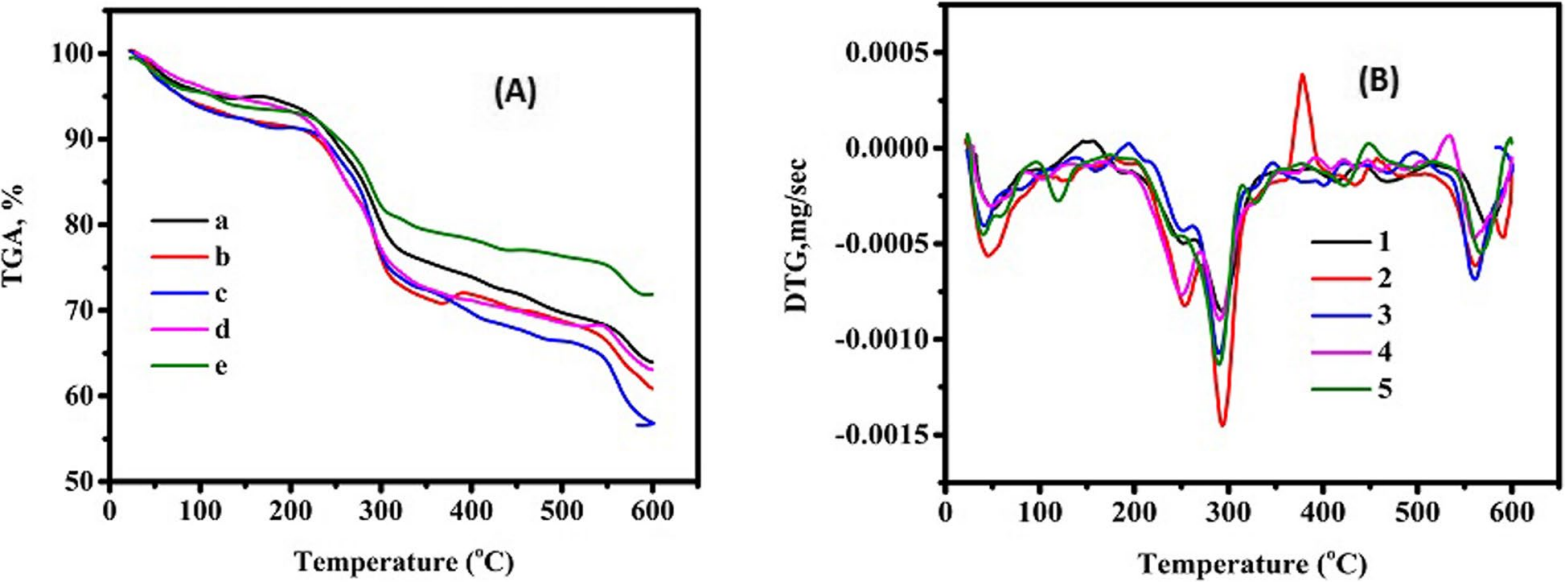
Thermogravimetric analysis spectra of un-irradiated and irradiated sodium alginate; (**A**) TGA% and (**B**) TDG, mg/sec. Also indicating that, 1 and a; control, 2 and b; irradiated 25 kGy, 3 and c; irradiated 50 kGy, 4 and d; irradiated 75 kGy, and 5 and e; irradiated 100 kGy

This technique may be utilized to better understand the thermal constancy as well as the thermal behavior of the UISA and ISA. Thermal gravimetric analysis, as shown in Table 2 and Fig. 3-(A) and (B), presents the TGA and TDG, respectively. The thermal gravimetric analysis curvatures and the weight loss percentage of UISA and ISA at various γ-ray dose levels. The weight loss occurred in three phases, as can be observed from the data. The first step of weight loss began under 50 ºC, and it was attributed to the loss of water molecules that cooperate with the hydroxyl and carboxylic groups of the Na-alginate structure via hydrogen, which was 50 ºC for UISA and 40 ºC for 100 kGy. The second one was the melting point was 293 ºC for UISA and 289 ºC for 100 kGy. The third one was that the oxidation point was 575 ºC and 567 ºC for the UISA and ISA, respectively, and this decomposition appeared among temperatures field 290–600 °C with the greatest corresponding breakdown of the saccharide ring in the molecule, as it is clear from Table 3 that the minimum weight reminder was in the ISA at 100 kGy (28.12%) compared to the UISA (43.39%).

**Table 2:** Remaining weight of un-irradiated and irradiated sodium alginate after TGA

Sample	Weight (%) at 600 °C	Reminder wt %
1	63.9382	36.0618
2	60.88234	39.11766
3	56.60125	43.39875
4	63.06436	36.93564
5	71.87439	28.12561

**Table 3:** Influence of un-irradiated Na-alginate (UISA) and irradiated Na-alginate (ISA) at dose level 100 kGY different concentrations on growth parameter of lettuce plant

Concentrate of SA (ppm)		Leaf area (cm^2^)	Stem length (cm^2^)	Head weight (g/plant)	Leaves number
H_2_O		279 ± 1.44^d^	20.30 ± 0.64^b^	305.20 ± 6.64^c^	33.00 ± 1.15^e^
UISA	200	284.2 ± 4.35^ cd^	22.81 ± 1.79^b^	329.30 ± 9.93^b^	36.00 ± 1.15^ cd^
ISA	200	299.1 ± 2.3^b^	23.32 ± 2.02^b^	335.18 ± 8.63^b^	39.00 ± 1.73^bc^
	400	316.6 ± 1.04^a^	28.10 ± 1.34^a^	373.91 ± 2.48^a^	43.00 ± 1.15^ab^
	600	300.7 ± 2.71^b^	30.32 ± 1.39^a^	384.20 ± 4.62^a^	46.67 ± 1.76^a^
	800	293 ± 2.54^bc^	23.60 ± 1.04^b^	345.10 ± 3.18^b^	37.00 ± 1.16^ cd^

Results are uttered as averages ± SE (*n* = 3), and means by different letter exclusive the same column are significantly difference (*p* ≤ 0.05)

The change in greatest temperature after SA irradiation in both of the breakdown phases might be due to the breakdown of the alginate intermolecular chain. A significant quantity of water is gone prior to 150 °C owing to the hydrogen bond of the alginate backbone, when it is linked to -OH and COO- groups, and also is in the range of 220 °C to 300 °C, which contributes to the division of sodium alginate, but the residue remains pure sodium alginate powder owing to the creation of sodium oxide once the temperatures are greater than 500 °C [30]. Weight loss is related to the evaporation of the water from the surface, and by raising the temperature, additional loss is due to the oxygen release from the samples [31]. There are noticeable changes to the thermal stability of gamma-irradiated sodium alginate at various dose levels [32].

### Scanning electron microscope (SEM)

**Fig. 4:**
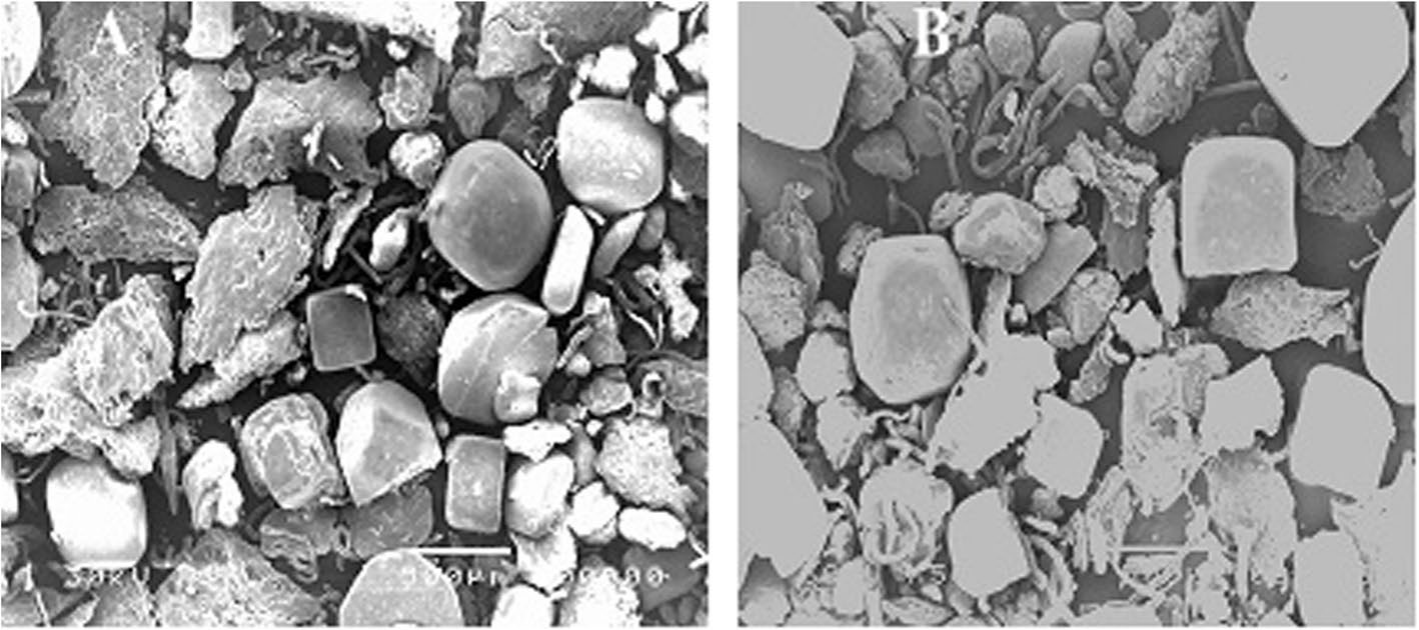
Scanning electron microscopy (SEM): un-irradiated sodium alginate (**A**), γ-irradiated Na-alginate at dose level 100 kGy (**B**)

The SEM images of un-irradiated and γ-irradiated Na-alginate (100 kGy) are provided in Fig. 4, which shows the morphology structure of UISA (A) and ISA (B) by scanning electron microscope images. It is evident that the surface of sodium alginate is rough; it appears rock-like, and there was a clear reduction in particle sizes of Na-alginate after gamma-irradiation. Reducing the particle sizes of ISA compared with UISA treatments is due to the splitting of glycosid bonds.

### Transmission electron microscope (TEM)

**Fig. 5:**
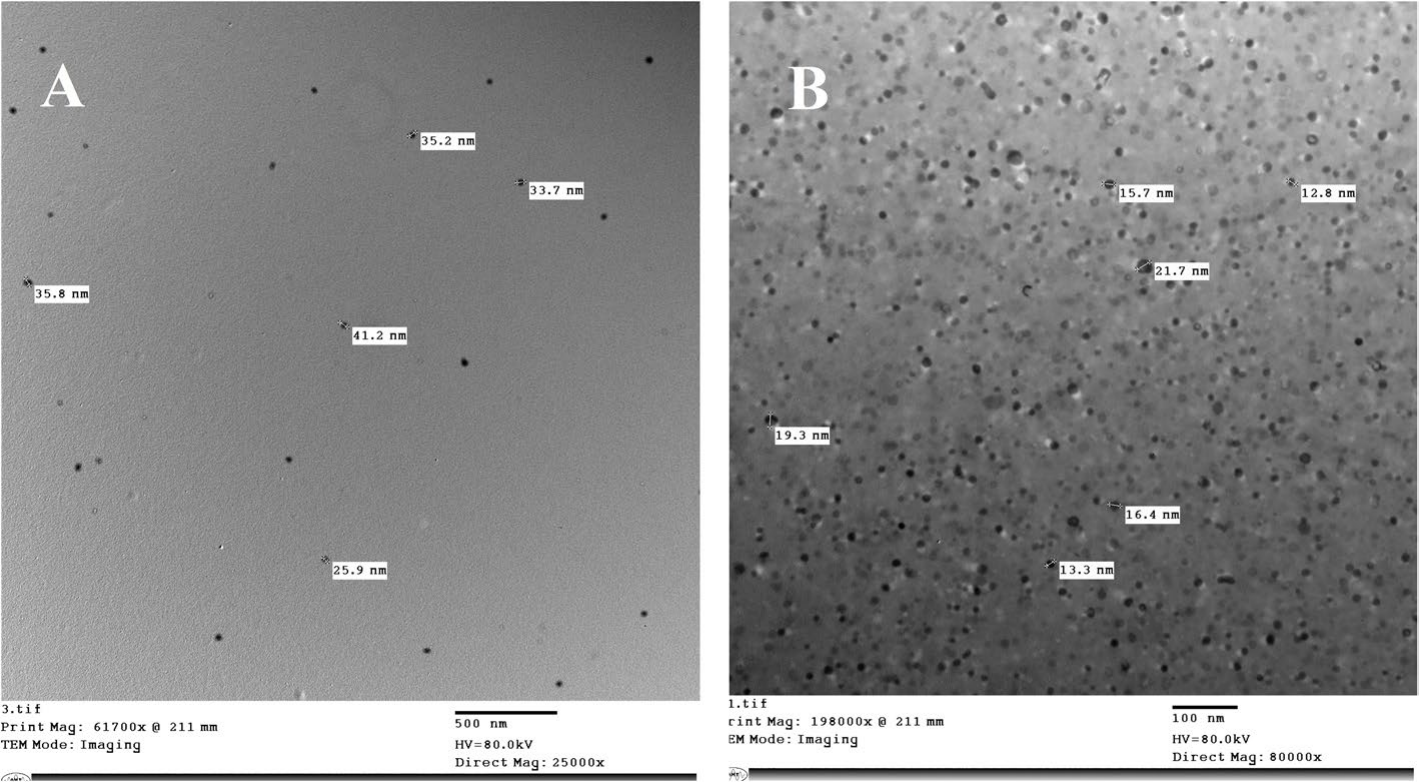
Transmission electron microscope (TEM) for un-irradiated Na-alginate (**A**), γ-irradiated Na-alginate (**B**) at dose level of 100 kGy

**A** transmission electron microscope (TEM) was applied to study the morphology and size of un-irradiated and irradiated sodium alginate. The images, as outlined in Fig. 5, describe that the Na-alginate is spherical in shape, with a particle distribution between 12.8 and 21.7 nm in ISA. While the particle size of UISA ranged between 25.9 and 41.2, showing a reduction of 46.7% in the particle size due to irradiation. The recent data are in line with Shabbir et al. [9], who established that the sodium alginate pore size decreased by increasing the γ-irradiation dose level up to 100 kGy, the macromolecular structure disappeared, and the surface became smoother.

### Growth parameters

Polysaccharides such as sodium alginate have been successfully used as plant growth-promoting substances in their depolymerized form. Gamma-ray degrades the sodium alginate into smaller oligomers with a comparatively low molecular weight. Application of these oligomers to plants resulted in various biological and physiological activities, including the promotion of plant growth, including seed germination, shoot elongation, root growth, and flower production [22]. Gamma-irradiated Na-alginate (100 kGy) has numerous novel unique features that can be beneficial in agronomy. As cleared in Table 3, ISA stimulated the growth parameters of the lettuce plant as the leaf area (316.6 cm^2^), was enhanced with the concentration (400 ppm), as well as stem length, head weight, and leaf number, which increased with the concentration (600 ppm) compared to the control (H_2_O treatment) and un-irradiated sodium alginate (Table 3).

Several studies have confirmed that irradiated Na-alginate efficiently acts as a plant growth agent and also enhances the activities of numerous enzymes in plants [18]. Gamma-irradiated sodium alginate induces plant defense responses and biological activities in plants. It was noticed that the application of ISA appeared to have a noteworthy effect on the plant height and leaf number, and it was observed that the maximum plant height and leaf number average of malabar spinach was found when treated with irradiated sodium alginate. Otherwise, the same strains decreased when treated with water (control sample) and an un-irradiated Na-alginate solution. [25]. Depolymerization of sodium alginate (powder form) requires high irradiation doses and a change in the molecular weight of alginate as a result of radiation-based degradation. Furthermore, it is speculated that after gamma irradiation, these oligosaccharides bind to certain receptor proteins, inducing different signaling pathways and thereby eliciting improved growth and yield in plants [27]. It was also found that alginate oligomers have a functional resemblance to endogenous growth elicitors and may act as signaling molecules, which apparently activate the plant’s responses by stimulating gene expression and synthesizing various enzymes [9]. In the same concern, Idrees et al. [34] exhibited that after gamma irradiation of sodium alginate, the distribution curve showed the elution of different molecular weight fractions. Since these fractions are small enough to penetrate easily through the stomatal aperture upon foliar feeding, they are expected to trigger growth-promoting activities in plants. The obtained results agree with previous studies, which showed the depolymerization of sodium alginate greatly affected plant vegetative traits depending on the level of depolymerization as a result of ISA. The growth and development of barley and soya bean increased when treated with ISA of molecular weight from 1 to 3 kDa [35]. Moreover, it was noted that depolymerized polysaccharides, including alginate, carrageenan, and chitosan, have unique features such as promoting germination and shoot elongation in sugarcane [36]. Treating plants with irradiated sodium alginate showed a considerable improvement in growth-related, physiological, and biochemical properties, as well as promoting the main components and yields of *Eucalyptus citriodora,* and that is owing to the degraded alginate, which improved water and mineral uptake by roots and thus increased the level of nutrients in plant tissue [37]. Comparable growth indicators were also obtained from the irradiated Na-alginate treatment on numerous crops, irrespective of plant categories, e.g., fruits, vegetables, oil, or legume-producing plants [12, 38, 39].

### Photosynthetic pigments (mg/g FW)

The effects of UISA and ISA (100 kGy) on the chlorophyll contents of lettuce plants are illustrated in Table 4. It was observed that the addition of ISA up to 400 ppm significantly increased Chl a, Chl b, carotenoid, and total pigment concentrations of 0.752, 1.069, 0.209, and 02.209 mg/g FW, respectively. While increasing the concentration of ISA to 800 ppm, it decreased all these contents compared to un-irradiated sodium alginate. Foliar application of irradiated Na-alginate improved photosynthetic parameters, and the total chlorophyll and carotenoids reached to maximal values after the application of ISA compared to the control [9]. The highest increase in pigments was observed in ISA-treated plants; therefore, ISA-treated plants could trap more sunlight to increase the rate of photosynthesis compared to the control plants [22]. The use of γ-irradiated Na-alginate might have enhanced photosynthetic efficiency through efficient assimilation and translocation [27]. Moreover, small alginate molecules had higher stomatal uptake, enhanced the photosynthesis, and thus increased the leaf size [40]. A stimulating the effect of sodium alginate on chlorophyll content may be due to increase leaf nitrogen, which is a constituent of the chlorophyll molecule. This stimulating effect on total chlorophyll and carotenoid is also observed in the opium poppy plant [41]. The increased levels of photosynthetic pigments after the application of ISA may be due to a potential increase in the number and size of chloroplasts and the chlorophyll content per chloroplast. It might also be due to the enchantment of water and nutrient uptake from the soil, followed by the smooth translocation of photosynthesis and additional metabolites [42]. The concentration of irradiated Na-alginate depends on the γ-source and the units (kGy) of radiation for certain plants [43]. Furthermore, the current findings are consistent with the previous results, which showed irradiated caragenan-mediated enhancement in photosynthetic parameters [44, 45]. The higher photosynthetic rate brought by the foliar application of ISA may have been caused by the increased content of photosynthetic pigments, stomatal conductance, enhanced rubisco, and CO_2_ fixation, which in turn increased phosphorylation processes [39]. An improvement in physiological attributes might be due to the ISA-mediated stimulation of gene expression, leading to an improvement in enzymatic activities, photosynthetic pigments, and the net photosynthetic rate [9]. Irradiated Na-alginate improved the leaf area, which was attributed to the utilization of more carbon dioxide by the leaves, which enhanced photosynthesis and the removal of more dry matter while receiving more sunlight [46].

**Table 4:** Influence of un-irradiated Na-alginate (UISA) and γ-irradiated Na-alginate (ISA) at dose level of 100 kGy different concentrations on photosynthetic pigments

Concentrate of SA (ppm)		Chl a (mg/g FW)	Chl b (mg/g FW)	Carotenoids (mg/g FW)	Total pigments (mg/g FW)
H_2_O		0.234 ± 0.034^e^	0.533 ± 0.025^c^	0.138 ± 0.008^c^	0.904 ± 0.087^e^
UISA	200	0.306 ± 0.057^d^	0.6377 ± 0.038^c^	0.140 ± 0.009^c^	1.08 ± 0.086^d^
ISA	200	0.416 ± 0.083^c^	0.830 ± 0.059^b^	0.169 ± 0.027^b^	1.41 ± 0.037^c^
	400	0.752 ± 0.073^a^	1.069 ± 0..097^a^	0.209 ± 0.026^a^	2.029 ± 0.096^a^
	600	0.588 ± 0.38^b^	0.920 ± 0.063^b^	0.181 ± 0.017^b^	1.689 ± 0.087^b^
	800	0.247 ± 0.047^e^	0.631 ± 0.566^c^	0.1665 ± 0.028^b^	1.045 ± 0.047^d^

Results are uttered as averages ± SE (*n* = 3), and means by various letter inside the same column are significantly difference (*p* ≤ 0.05)

### Correlation between morphological traits and total pigments

**Fig. 6:**
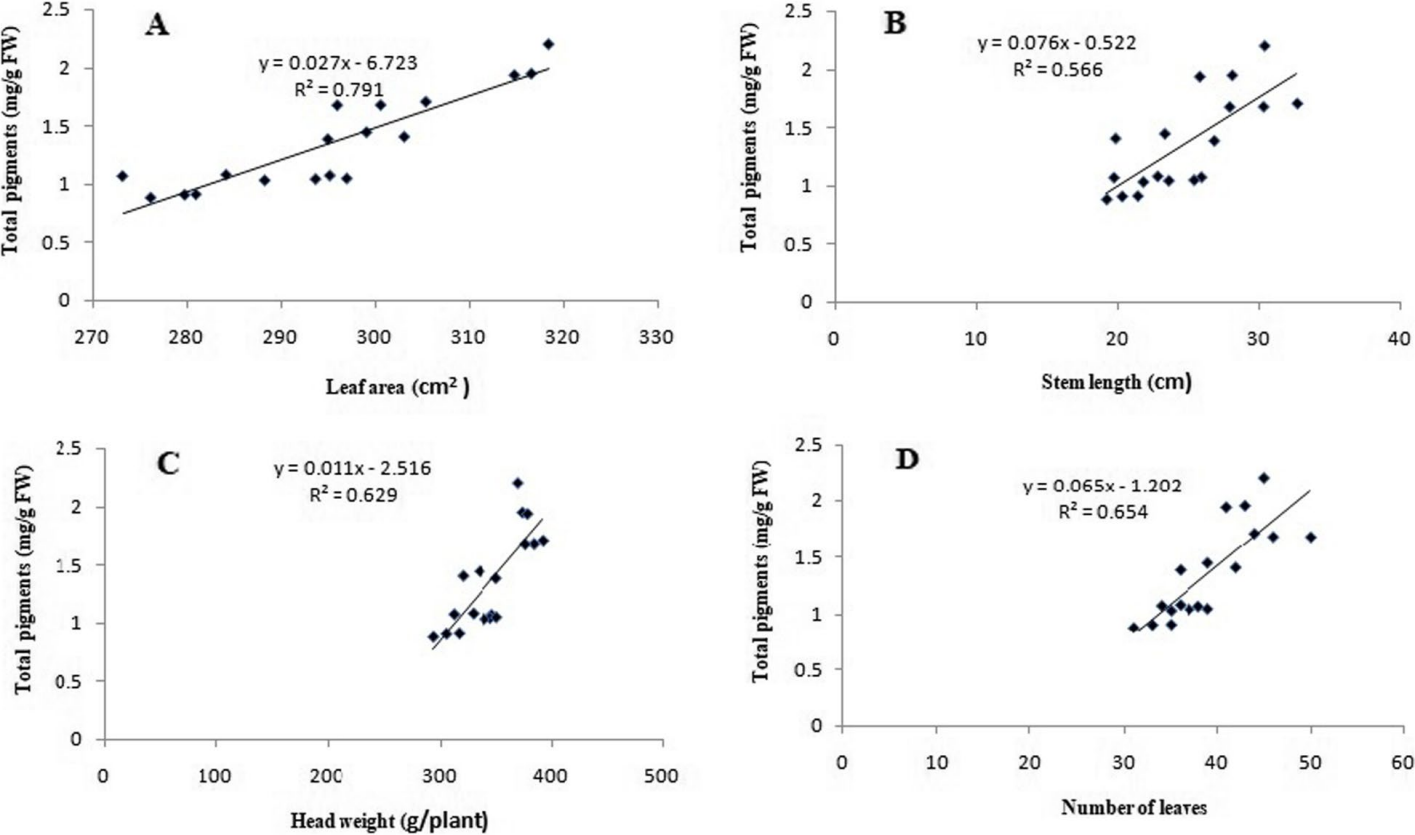
Correlation graphs for lettuce plant, total pigments (mg/g FW) *vs.* leaf area (cm^2^ A), stem length (cm B), head weight (g/plant C) and leaf numbers (D) under un-irradiated and irradiated sodium alginate (100 kGy) different concentrations

A quantitative analysis was also used to investigate the correlation between total pigments and morphological traits such as leaf area, stem length, head weight, and leaf numbers. Four correlation graphs [total pigments *vs.* leaf area (A), stem length (B), head weight (C), and leaf numbers (D)] were illustrated in Fig. 6. The total pigment content showed a significant positive correlation to leaf area, number of leaves, head weight, and stem length, with Pearson’s correlation coefficients of 0.790, 0.654, 0.629, and 0.566, respectively. These results indicated that the morphological traits under study significantly contributed to the total pigment of lettuce leaves due to the irradiated Na-alginate treatment. The correlation coefficients are naturally indicating the strength of the relationships between these studied factors. In the same concern, Li et al. [47] reported that the oligosaccharides can interact with cells as signaling molecules or act as inducers to regulate the growth pattern of plants and effectively regulate physiological activities related to the antioxidant system, photosynthesis, and nutrient uptake, triggering the synthesis of different enzymes and activating various responses via changes in gene expression. A study has also confirmed that photosynthesis affects the area of the leaf to varying degrees and has an impact on photosynthetic properties such as photosynthetic rate and carbon fixation [48].

### Total phenolic, flavonoid contents and antioxidant activity

**Fig. 7:**
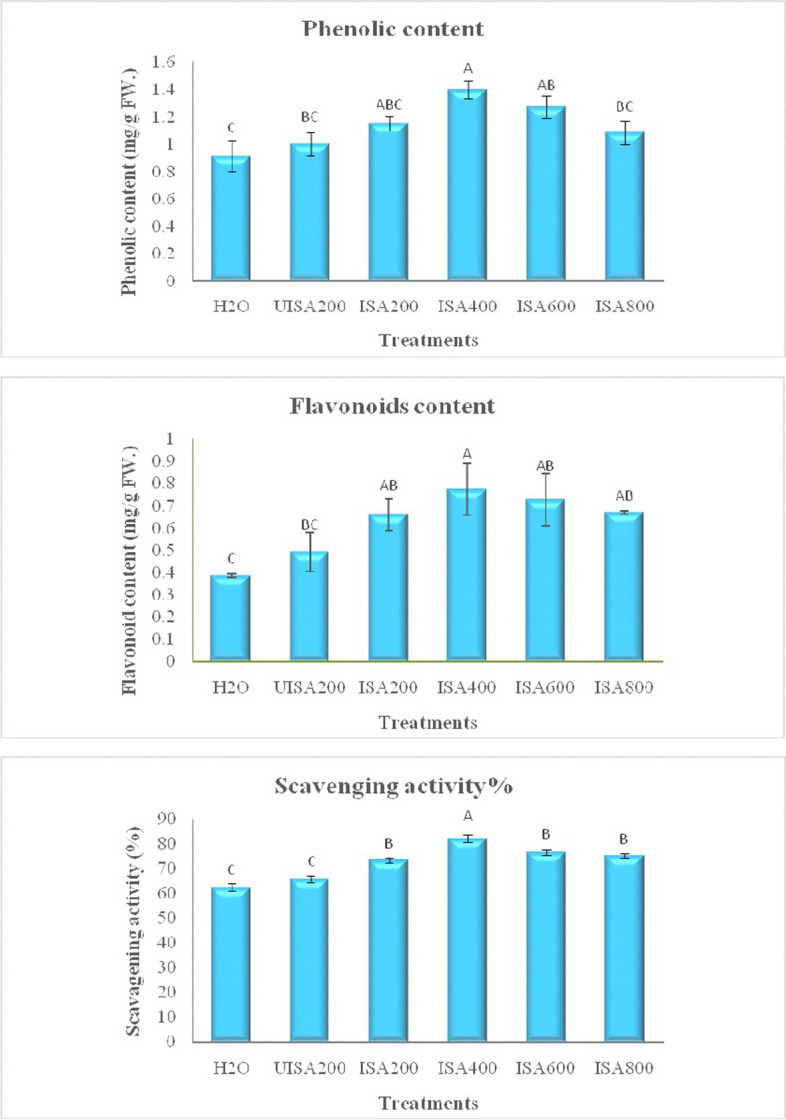
Effect of un-irradiated Na-alginate (UISA) and γ-irradiated (100 kGy) Na-alginate (ISA, ppm) on total phenols total flavonoids contents (mg/g FW) and antioxidant activity (%) of lettuce plant. Results are expressed as means and vertical bars ± SE (*n* = 3) and varying letters above bars in each sample are significantly difference at (*p* ≤ 0.05)

Findings in Fig. 7 clearly show the impact of un-irradiated and irradiated Na-alginate on the phenolic content of the lettuce plant. It is notable that irradiated sodium alginate (400 ppm) significantly gave the highest value of phenolic compounds (1.399 mg/g FW) among all treatments used and controls.

Flavonoid content was significantly affected by irradiated sodium alginate, as shown in Fig. 7. It demonstrated that irradiated sodium alginate (400 ppm) significantly increased flavonoid content (0.7748 mg/g FW).

Results in Fig. 7 show both un-irradiated and irradiated sodium alginate on antioxidant scavenging activity. In comparison to the other treatments, it is verified that irradiated sodium alginate enhanced DPPH scavenging activity to the greatest rise with 400 ppm ISA (84.47%). The application of irradiated Na-alginate at 100 mg/liter raised the total phenolic content in the leaves of *Mentha arvensis* L [49]. The positive effect in response to irradiated Na-alginate application might be attributed to the specific role of oligomers divided by gamma-ray application in plants [18]. Meanwhile, there is an increase in the secondary metabolites such as total phenols content, total flavonoids, lipid peroxidation, and antioxidant activity of safflower callus through elicitation at various levels of Na-alginate (0.075 and 0.15%) [50]. Otherwise, the application of oligomeric fractions to plants resulted in a significant improvement in quality, leaves with more pigments, polyphenols, and ascorbic acid content, as well as they also exhibited slightly increased antioxidant activity [51]. Gamma-irradiated Na-alginate on the dose level 25 kGy enhanced growth parameters, photosynthetic contents, and the concentrations of MDA, proline, and total phenols when compared to controls of mung bean (*Vigna radiata* L.) [52]. Meanwhile, Li et al. [47] indicated that alginate oligosaccharides, when sprayed on leaves, (spray application) may rapidly trigger the activation of the antioxidant system and increase the activity of antioxidant enzymes, which may assist the leaf survival conditions. On the other hand, Naeem et al. [49] reported that treatment with ISA significantly decreased total phenols, free proline, and MDA contents as compared to the control plants.

### Correlation between morphological traits and antioxidant activity

**Fig. 8:**
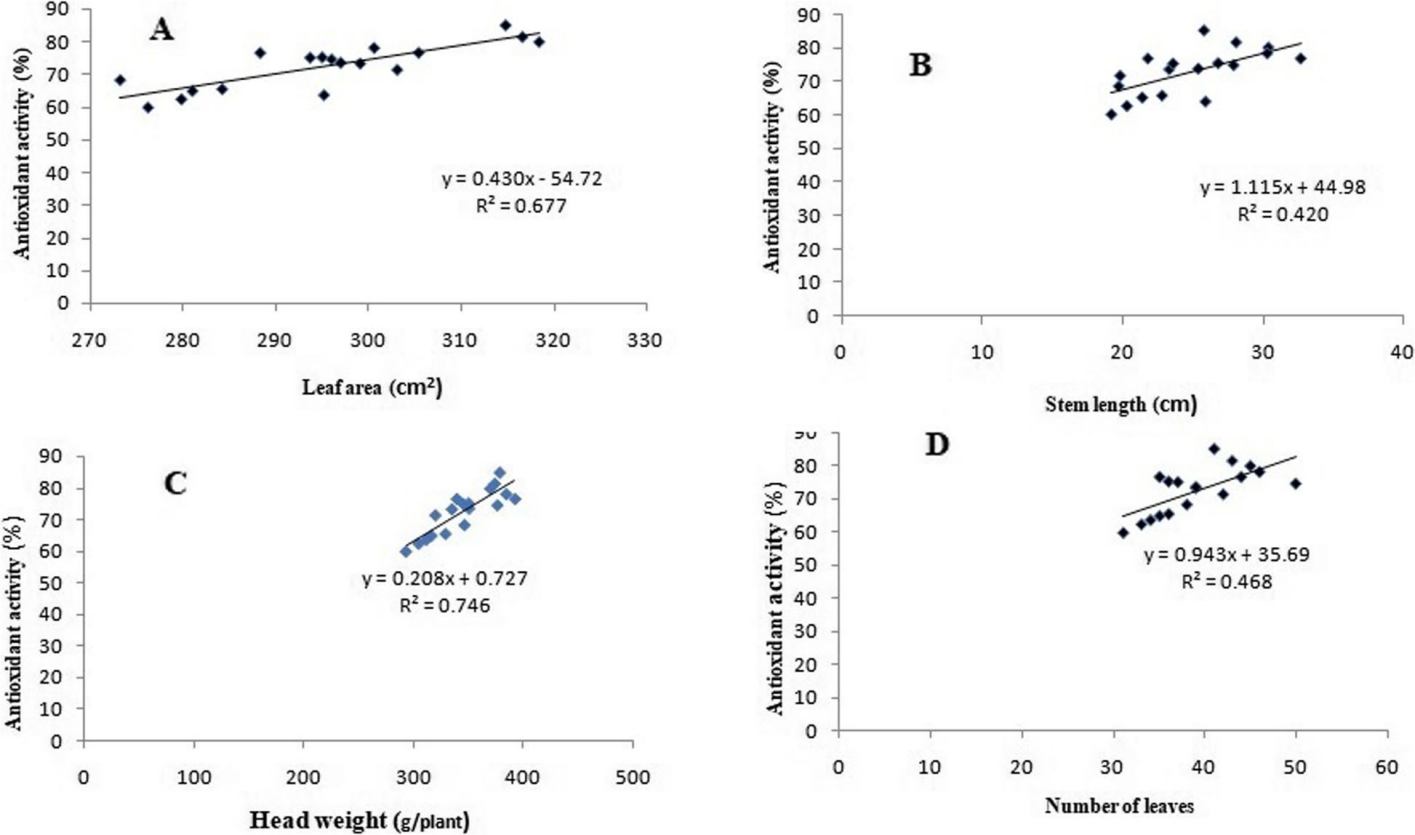
Correlation graphs for lettuce plant, antioxidant activity (%) *vs.* leaf area (cm^2^ A), stem length (cm B), head weight (g/plant C) and leaf numbers (D) under irradiated sodium alginate (100 kGy) different concentrations

A quantitative analysis was also used to investigate the correlation between the percentage of antioxidant activity and morphological traits such as leaf area, stem length, head weight, and leaf numbers. Four correlation graphs [antioxidant activity *vs.* leaf area (A), stem length (B), head weight (C), and leaf numbers (D)] were illustrated in Fig. 8. The antioxidant activity provided a significant positive correlation to head weight, leaf area, number of leaves, and stem length, with Pearson’s correlation coefficients of 0.746, 0.677, 0.468, and 0.420, respectively. According to these findings, the ISA treatments significantly increased the examined morphological features' antioxidant activity in lettuce leaves.

The degree of the correlations among these investigated parameters is intuitively denoted by the correlation coefficient. Head weight and leaf area were more correlated to the antioxidant activity than stem length and leaf numbers. Meanwhile, Chiappero et al. [53] reported that plant growth promoters might improve the quality of plant growth, resulting in primary metabolites as the predominant metabolic product. Primary metabolites are considered to be a strong source for antioxidant activity in plants.

### Total soluble proteins and ascorbic acid (mg/g FW)

**Fig. 9:**
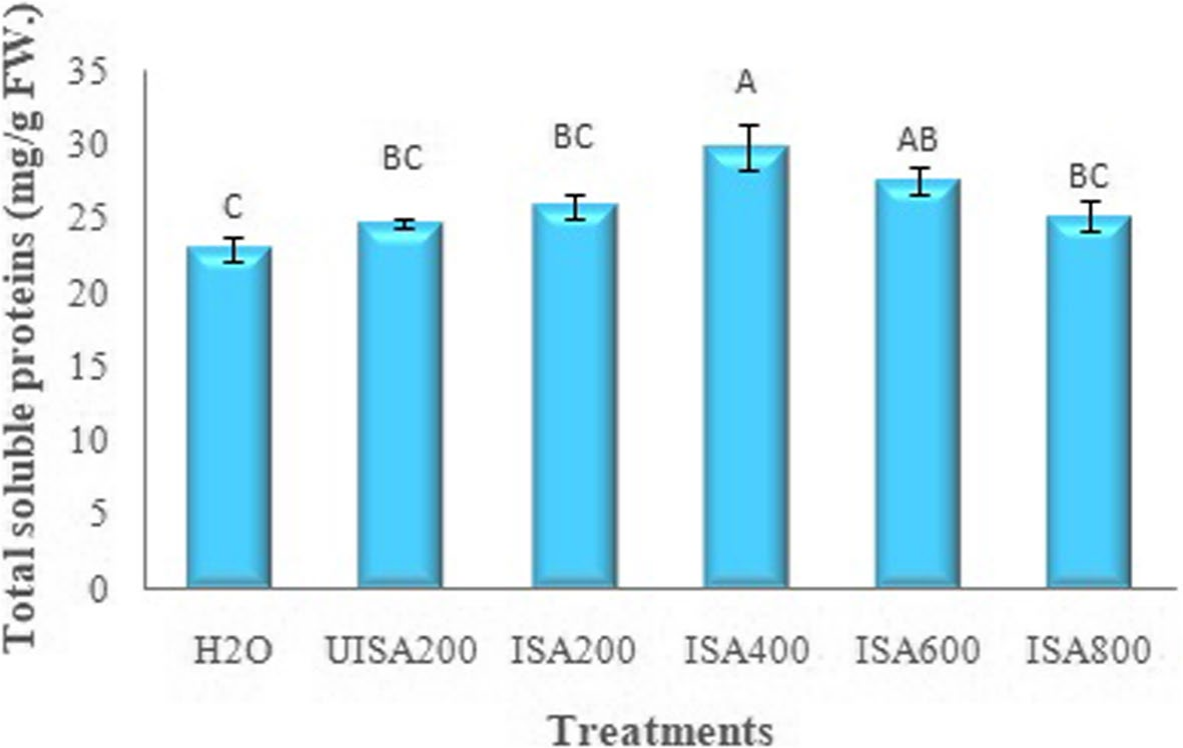
Effect of un-irradiated Na-alginate (UISA) and γ-irradiated (100 kGy) Na-alginate (ISA, ppm) on total soluble proteins (mg/g FW) of lettuce plant. Results are expressed as means and vertical bars ± SE (*n* = 3) and varying letters above bars in each sample are significantly difference at (*p* ≤ 0.05)

The effect of un-irradiated Na-alginate (UISA) and γ-irradiated (100 kGy) Na-alginate (ISA) on the total soluble protein content of the lettuce plant is provided in Fig. 9. It is obvious that irradiated sodium alginate (400 ppm) gave the highest content of total soluble protein (29.96 mg/g FW) compared to all the treatments used.

**Fig. 10:**
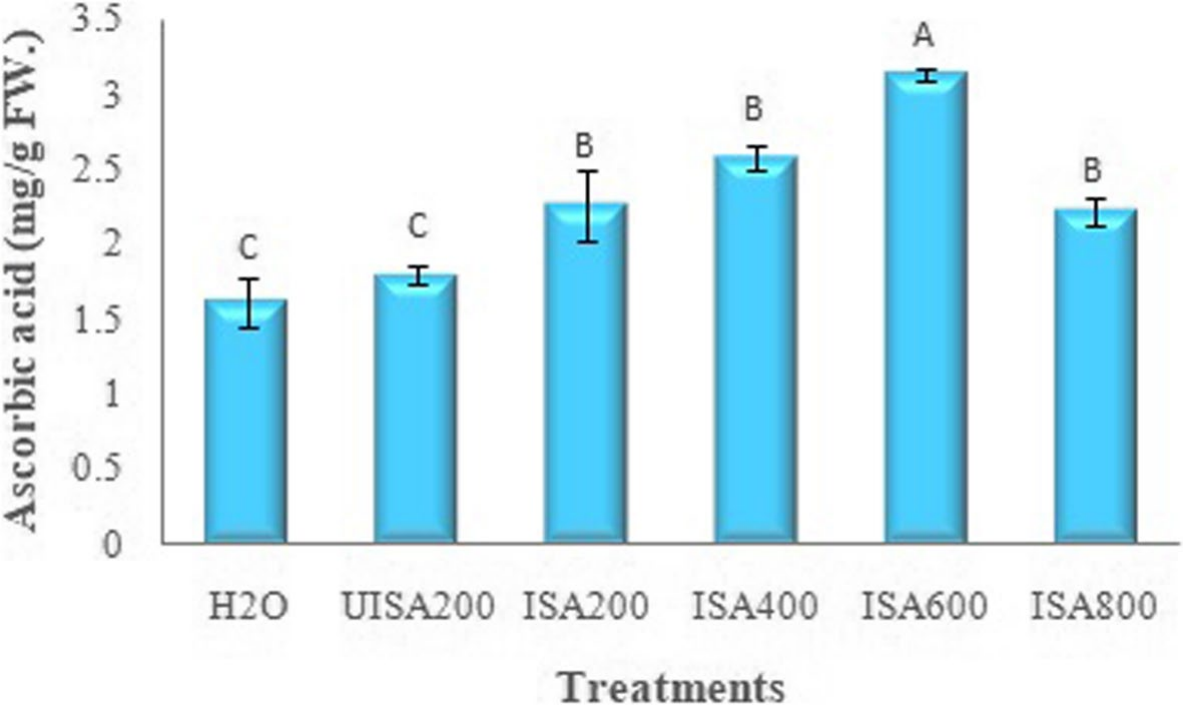
Effect of un-irradiated Na-alginate (UISA) and γ-irradiated (100 kGy) Na-alginate (ISA, ppm) on ascorbic acid (mg/g FW) of lettuce plant. Results are expressed as means and vertical bars ± SE (*n* = 3) and varying letters above bars in each sample are significantly difference at (*p* ≤ 0.05)

Concerning ascorbic acid content, as observed in Fig. 10, when ISA increased its content, a maximum increase was observed at 400 ppm (3.10 mg/g FW), followed by 600 ppm (2.58 mg/g FW), as compared with water (1.62 mg/g FW) and un-irradiated sodium alginate (1.80 mg/g FW).

The increase in osmoregulation and soluble protein in castor beans (*Ricinus communi*) is attributed to the irradiated carrageenan. The irradiated Na-alginate treatment on pineapple showed a slightly higher content of vitamin C compared to the untreated control [54]. The ascorbic acid oxidase enzyme (ascorbinase) is in fact directly responsible for increasing vitamin C contents [38]. Moreover, the total polyphenol content, L-ascorbic acid, and antioxidative activity increased in the *Eucomis autumnalis* plant covered by oligoalginate compared to the lowest values in the untreated plant [50]. The promoting effect of oligoalginate on L-ascorbic acid as well as antioxidant activities under salinity stress reflects the protection of this substrate. Sodium alginate derivatives alter the production of certain substances containing active components and enhance plant metabolites [18, 55].

### Correlation between morphological traits and total soluble proteins

**Fig. 11:**
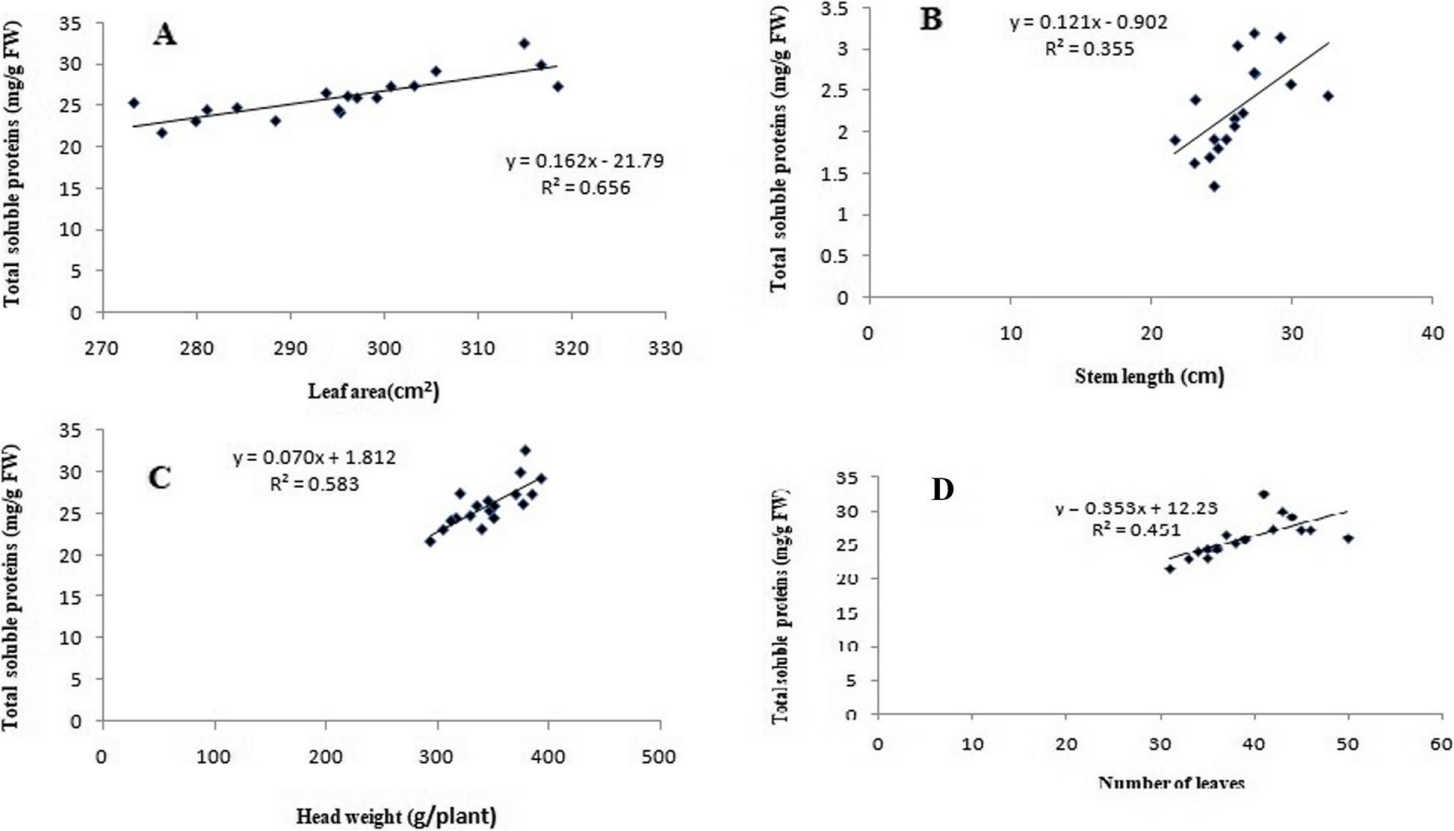
Correlation graphs for lettuce plant, total soluble proteins (mg/g FW) *vs.* leaf area (cm^2^ A), stem length (cm B), head weight (g/plant C) and leaf numbers (D) under irradiated sodium alginate (100 kGy) different concentrations

The correlation analysis data showed positive relations among the total soluble proteins and the morphological traits such as leaf area, stem length, head weight, and leaf numbers as provided in Fig. 11. Four correlation graphs [total soluble proteins *vs.* leaf area (A), stem length (B), head weight (C), and leaf numbers (D)] are declared in Fig. 11. Total soluble proteins showed a significant positive correlation to leaf area, head weight, number of leaves, and stem length, with Pearson’s correlation coefficients of 0.656, 0.583, 0.451, and 0.355, respectively. In particular, obviously, the leaf area had significantly the highest correlation with total soluble proteins. Parvin et al. [25] suggested that foliar spraying of irradiated sodium alginate at certain concentrations showed a marked effect on the height of plants and the number of leaves, as well as causes an increase in the biochemical and physiological functions of the spinach plant. Furthermore, protein degradation and recycling are essential responses of plants to stress, since the breakdown of proteins generates free amino acids needed for the de novo synthesis of new proteins [56].

### Correlation between morphological traits and ascorbic acid

**Fig. 12:**
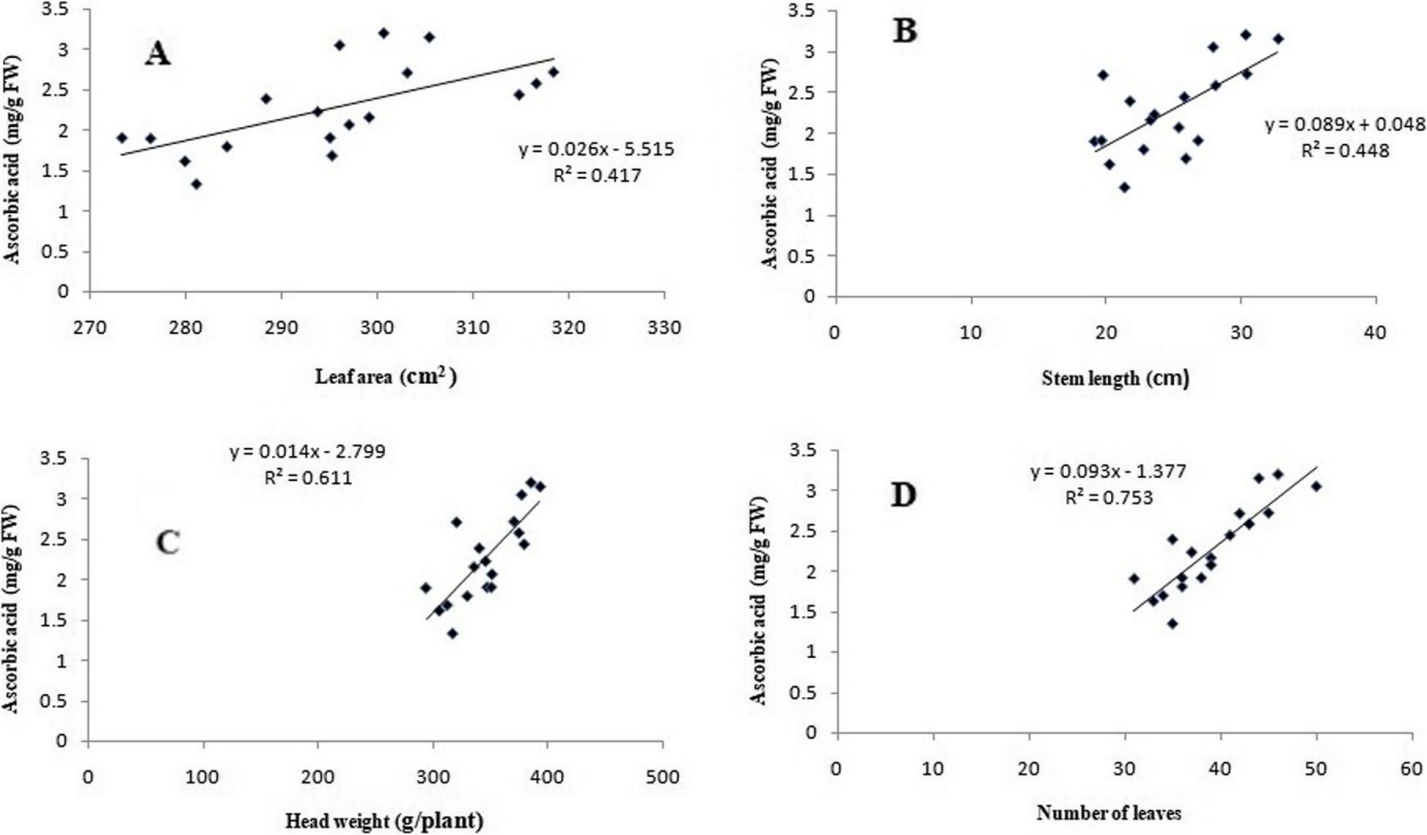
Correlation graphs for lettuce plant, ascorbic acid (mg/g FW) *vs.* leaf area (cm^2^ A), stem length (cm B), head weight (g/plant C) and leaf numbers (D) under irradiated sodium alginate (100 kGy) different concentrations

The correlation analysis results indicated positive relations between the ascorbic acid and morphological traits such as leaf area, stem length, head weight, and leaf numbers. Correlation graphs [ascorbic acid *vs.* leaf area (A), stem length (B), head weight (C), and leaf numbers (D)] were stated in Fig. 12. Ascorbic acid provided a significant positive correlation to the number of leaves, head weight, stem length, and leaf area, with Pearson’s correlation coefficients of 0.753, 0.611, 0.448, and 0.417, respectively. Particularly, the number of leaves significantly provided the maximum correlation with ascorbic acid. Vitamin C is a vital element of plant and animal antioxidant systems, which can be defined as complex redox networks, including metabolites and enzymes, with mutual interactions and synergistic effects [57]. Moreover, vitamin C content varies among different tissues and organs, usually being high in leaves, meristematic tissues, flowers, or young fruits and low in non-photosynthetic organs such as stems and roots [58]. Also, in plants, vitamin C can control the division, elongation, and differentiation of cells, as well as programmed cell death (PCD). Vitamin C plays a significant role in the control of cell division [59].

## Conclusion

In conclusion, gamma irradiation exposure results in the depolymerization of sodium alginate into low-molecular-weight oligosaccharides. Depolymerization also leads to certain structural changes, which cause the oligosaccharides to elicit various beneficial responses in plants with regard to growth, yield, and quality. Sodium alginate is fully harmless to the environment and has a wide range of uses. The growth-enhancing effect of gamma-irradiated Na-alginate was established to be greater than that of the un-irradiated alginate, attributed to the achievement of definite structural modification and a decrease in particle size brought by gamma-irradiation. The current investigation exposed that foliar application of irradiated Na-alginate was significant in promoting growth, physiological, and biochemical characteristics. This has proven surprisingly effective in improving the composition and yield of the lettuce plant. It could be concluded that applying irradiated Na-alginate could be beneficial as a plant growth promoter in the future. The use of sodium alginate may potentially be utilized as a plant growth enhancer to achieve desirable qualities in multiple ways to reach the preferred superiority of numerous crops and plants. The greatest value with regard to the whole performance of the plants was gained by applying irradiated Na-alginate at 400 ppm, followed by 600 ppm. Furthermore, the correlation coefficients (R values) of total pigment content, antioxidant activity, total soluble proteins, and ascorbic acid with leaf area, stem length, head weight, and leaf number values also showed a positive correlation. Therefore, primed natural growth promoters can play a vital role in meeting the promising food needs. However, further studies are required to comprehend the mechanism and mode of action of Na-alginate-derived oligomers on the productivity and quality of plants and agricultural crops.

## Material and method

### Irradiation treatment

Sodium alginate was degraded by exposing it in powder form to gamma-rays (^60^Co Gamma cell 220-Canada) at different dose levels (25, 50, and 100 kGy) with a rating dose of 1.1 kGy/h at the time of irradiation. The irradiation handling has been achieved at the Egyptian Atomic Energy Authority (EAEA), National Center for Radiation Research and Technology (NCRRT), Cairo, Egypt.

### Physicochemical characterizations of sodium alginate

#### Fourier transform infrared (FTIR) spectroscopy

The FTIR of un-irradiated and irradiated sodium alginate has been performed using FTIR spectroscopy (Bruker, Unicom, Germany) at a resolution of 4 cm^−1^ in the 4000–500 cm^−1^ wave number range.

### X-ray diffraction (XRD)

X-ray diffraction measurements of un-irradiated and γ-irradiated sodium alginate were obtained by the XD-DI g (Shimadzu Diffractometer D6000, Japan) using nickel-filtered and Cu-K targets. The XRD has been carried out over the 2θ range from 4° to 90° at a scan speed of 8 °C/min, 30 mA, and a voltage of 40 kV.

### Thermogravimetric analysis (TGA) determination

The TGA thermograms of un-irradiated and irradiated sodium alginate have been performed by the Shimadzu 50 instrument (Kyoto, Japan) with a heating rate of 10 ºC/min and a nitrogen flow rate of 20 ml/min at ambient temperature to 600 ºC.

### Scanning electron microscope (SEM)

The surface of the sample was examined using a Jasco JSM-5200 scanning electron microscopy (SEM), Japan, by voltage acceleration at 25 kV after gold eposition in vacuum for three minutes [60].

### Transmission electron microscopy (TEM)

The structure of the nanocomposites was observed by transmission electron microscopy (JEOL-JEM 1400CX electron microscope, Japan) at an accelerating voltage of 80 kV. Sodium alginate samples were prepared by gentle grinding and mixed with acetone, followed by a sonication procedure. The solution was deposited in one drop on a microgrid to avoid the high-voltage electron beam from damaging the taster; the chamber of the taster inside the device was put in a bath of liquid nitrogen to alleviate the temperature [61].

### Experiment design

This experiment was conducted in the greenhouse belonging to the Natural Products Research Department, NCRRT, and a randomized design was carried out. As a result of the physicochemical characterizations of sodium alginate with different dose levels (25, 50, 75, and 100 kGy) and found that the irradiated sodium alginate with 100 kGy is the most appropriate for the current investigation. Different concentrations of irradiated sodium alginate at dose levels of 100 kGy (200, 400, 600, and 800 ppm, as well as deionized water used as a control). Double-distilled water (DDW) was used to dissolve the UISA and ISA, and then various concentrations of 200, 400, 600, and 800 ppm were prepared using DDW. Un-irradiated sodium alginate was also dissolved in DDW and used at a concentration of 200 ppm, while DDW was used as a control.

Egyptian iceberg lettuce 21-day seedlings were planted in the experimental farm, where they were placed 50 cm apart and 40 cm between plants on both ends of the rows. Around thirty days old, the seedlings were first exposed to the UISA and ISA solutions. Eight spray treatments were applied at an interval of one week by hand sprayer, and data were recorded on five randomly picked plants from every plot from each of the three replicates. Plants were weeded and watered as needed, and all environmental and agricultural needs were met [62], as well as the chemical and physical properties of the experimental soil, as displayed in Table 5.

**Table 5:** Soil properties of the investigation site

Soil properties	
pH( 1:1)	7.32
EC ( 1:1) dS/m	2.2
Soluble anions (meq/l)	
CO_3_^=^	1.5
HCO_3_^−^	4.5
Cl^−^	8.0
SO_4_^=^	11.7
Soluble cations (meq/l)	
Ca^++^	10.5
Mg^++^	3.5
K^+^	0.50
Na^+^	11.2
Sand (%)	57.3
Silt (%)	21.2
Clay (%)	21.5
Texture class	Sandy clay loam

### Determination of growth traits

The growth attributes [leaf area (cm^2^), stem length (cm), head weight (g/plant), and number of leaves] were determined [63] at 70 days.

### Photosynthetic pigments

Chlorophylls a, b, and carotenoids of iceberg lettuce leaves have been evaluated spectrophotometrically by the method of von Wettstein [64]. Fresh leaf samples (0.5 g) were homogenized in a mortar with 85% acetone in the presence of washed dried sand and CaCo_3_ (0.1 g) to neutralize organic acids in the homogenate. The homogenate was then filtered through a sintered glass funnel. The residue was washed several times with acetone until the filtrate became colorless. The optical density of the obtained extracts was determined using a spectrophotometer (Jasco model V-530, Tokyo, Japan) at 662 and 644 nm for chlorophyll a and chlorophyll b, respectively, as well as 440.5 nm for carotenoids. Pigment contents were calculated in mg/g FW.

The following formula was used to determine the concentrations of carotenoids, chlorophyll a, and b:

$$\mathrm{Chlorophyll}\;a\hspace{0.17em}=\hspace{0.17em}9.784\hspace{0.17em}\times\hspace{0.17em}\text{A}662-0.99\hspace{0.17em}\times\hspace{0.17em}\text{A}644\;\mathrm{mg}/\text{l}$$

$$\mathrm{Chlorophyll}\;\mathrm{b}\hspace{0.17em}=\hspace{0.17em}21.426\hspace{0.17em}\times\hspace{0.17em}\text{A}644-4.65\hspace{0.17em}\times\hspace{0.17em}\text{A}662\;\mathrm{mg}/\text{l}$$

$$\text{Carotenoid}\hspace{0.17em}=\hspace{0.17em}4.695\hspace{0.17em}\times\hspace{0.17em}\text{A}440.5-0.268\mathrm{xc}\;(\text{a}\hspace{0.17em}+\hspace{0.17em}\text{b})\;\mathrm{mg}/\text{l}$$

### Ethanolic extract

Ethanolic extract was prepared by weighting two grams of fresh leaf sample, then ground with liquid nitrogen. After that, 25 ml of ethanol was included. The mix was vibrated for 24 h at room temperature. Whatman filter paper was used to filter the extract, and it was then extracted twice more [65]. After being adjusted to a specified volume, the resulting ethanolic extract was used to estimate total phenols, flavonoids, antioxidant activity, and total soluble proteins.

### Phenolic content

Total phenolic content in lettuce leaf extract treatments has been determined using the Folin-Ciocalteu technique, using gallic acid as a reference curve [66]. An aliquot of prepared ethanolic extract (0.5 ml) was mixed with 0.5 ml of Folin–Ciocalteau reagent, followed by the addition of 1.0 ml of sodium carbonate-saturated solution, and then completed with distilled water to 10.0 ml. This mixture was kept for 1 h at 25 °C in a dark place, and the absorbance value was read with a spectrophotometer (Jasco V-530, Tokyo, Japan) at 725 nm. The results have been stated as mg of gallic acid/g FW.

### Flavonoids content

The colorimetric assay with aluminum chloride was used to measure the total flavonoid content in leaf extract [67]. A sample (0.5 ml) of the ethanolic extract was mixed with 0.3 ml of 5% sodium nitrite. After that, 0.3 ml of 10% aluminum chloride was added 5 min later, followed by 2.0 ml of 1.0 M NaOH after 6 min and the total volume was completed to 5.0 ml with distilled water. The absorbance of the mixture was recorded at 510 nm by a spectrophotometer (JascoV-530, Tokyo, Japan) against the blank.. Outcomes were interpreted as mg quercetin equivalent (QUE)g^−1^ leaves fresh weight.

### Antioxidant activity

The antioxidant activities of the samples were performed against the radical 2,2- diphenyl-1 picryl hydrazyl (DPPH) [68]. Briefly, 0.1 mM of DPPH in methanol was prepared first, and 1.0 ml of this solution was added to 3.0 ml of iceberg ethanolic extract. The resulting mixture was vortexed and left at room temperature for 30 min in the dark. Absorbance was then conducted at 517 nm using a spectrophotometer (JascoV-530, Tokyo, Japan) against a blank. The following formula was used to calculate the radical scavenging activity:

$$\mathrm{DPPH}\;\mathrm{scavenging}\;\mathrm{activity}\;\left(\%\right)\hspace{0.17em}=\left[\left({\text{A}}_0-{\text{A}}_1/{\text{A}}_0\right)\right]\times100$$

Where,

${{\text{A}}}_{0}$ = The absorbance of control reaction (containing reagents except the test compounds)

A_1_ = The absorbance in the presence of the tested extracts.

### Total soluble proteins

Total soluble proteins were measured by the Bio-Rad microassay modification of the Bradford [69] procedure using crystalline bovine serum albumin as a reference.

### Ascorbic acid content

Vitamin C values were measured by the process used by Jagota and Dani [70]. Briefly, 0.2 g of fresh leaf samples were homogenated in 1.5 ml of 10% (w/v) TCA. The homogenates were then centrifuged at 3000 × g for five minutes, 0.3 ml of the extract was then taken and completed to two ml with DDW. After that, 200 µl of 10% (v/v) Folin-Ciocalteu was inserted into the mixture and forcefully vibrated. The maximum absorbance was read at 760 nm after ten minutes and the reaction result between the Folin reagent and L ( +)-ascorbic acid has been utilized as a reference. The outcomes were provided in mg vitamin C/g FW.

### Statistic analysis

Three replicates of each treatment were performed in a randomized full block design, and the outcomes were interpreted as average ± standard errors. Statistical analysis is analyzed using one-way analysis of variance (ANOVA). The analysis of the means value has been compared at *p* ≤ 0.05 with Duncan’s multiple rate tests [71].

## Data Availability

The data sets used and/or analyzed during the current study available from the corresponding author on reasonable request.
